# Son or Daughter Care in Relation to Self-Reported Health Outcomes for Older Adults in China

**DOI:** 10.3389/fpubh.2021.793873

**Published:** 2022-01-18

**Authors:** Yanan Zhang, Sarah Harper

**Affiliations:** Oxford Institute of Population Ageing, University of Oxford, Oxford, United Kingdom

**Keywords:** eldercare, health inequality, gender difference, self-reported health, CHARLS

## Abstract

**Objectives:**

Parental care in China is traditionally provided by sons. In recent decades—partly due to the One-Child Policy but also economic development and urbanization—significant changes have occurred with more and more parents receiving care from daughters. We investigate the disparities in outcomes of eldercare provided by son(s) and daughter(s).

**Methods:**

Our study compares the self-reported health (SRH) status of parents who receive eldercare from daughters and sons in China, analyzing the harmonized 2013, 2015, and 2018 waves of CHARLS with random-effects logistic estimates.

**Results:**

Our results show that the SRH status of parents who receive care from their sons is greater than those cared for by their daughters. This disparity is greater in rural areas, for mothers, and poorer families.

**Discussion:**

The One-Child Policy was more effective in urban areas, reducing both the availability of sons and cultural son preference. Higher levels of education received by girls in urban settings increases their employability and thus their ability to materially care for their parents. However, traditional norms and gender differences in social economic statuses still persist in rural areas, leading to higher SRH status of those cared for by sons, especially amongst those who are heavily dependent on their children: mothers or parents with less wealth.

## Introduction

In traditionally patrilineal societies such as China—influenced by the Confucian cultural norm—filial piety is valued as a core virtue, and married sons and daughters-in-law act as the primary caregivers to parents, while married daughters are expected to care for their husband's parents. Despite moves by the Chinese government to include daughters within the legal framework of care obligations, filial obligations remain most strongly with sons. Yet while traditional practices exclude married daughters from the filial discourse surrounding their own parents, they often have the most intimate relationship with their parents (1). Over recent decades, in part due to the One-Child Policy, but also arising from economic development and urbanization, significant changes have occurred in practice with daughters providing more and more support to their natal parents (2–5). Anthropological studies have suggested that the growing importance of parent-daughter relationships is specifically related to female independence and economic empowerment and the increased emphasis on affection and care replacing filial piety in parent-child relationships (6).

This has given rise to a particular interest in the contrasting provision of care (financial, emotional, or instrumental support for elderly parents) between daughters and sons (7–12). Changes in the gendered norm of sons providing care for elderly parents have been highlighted (13–15). As Lei (16) points out, there is a growing rural-urban divide in this respect as daughters provide more instrumental and emotional support to parents in urban China, whereas sons and daughters do not differ in rural China.

The literature on comparing the outcome of support provided by daughters and sons is limited. Zeng et al. (17) disclose that older parents whose primary emotional carer is a daughter (or son-in-law) are associated with a lower level of mortality rate and higher cognitive capacity than those with a son (or daughter-in-law) as primary carer. Zeng et al. (18) provide evidence that older parents are more likely to be satisfied with the support for activities of daily living (ADLs) provided by a daughter (or son-in-law) compared with the care given by a son (or daughter-in-law). Another study estimates that the likelihood of reporting an unmet need in ADLs support is lower among urban parents whose primary carer is a daughter than a son (19). Co-residence with a daughter was found to be associated with a higher level of mental wellbeing than co-residence with a son (20). The daughter advantage in providing care might be explained by the stereotyped gender norm that women are better at caring and the common conflicts between a daughter-in-law and a mother-in-law lowering parents' wellbeing (21).

However, there is also evidence showing the son advantage in providing instrumental care and affecting subjective well-being and mental health (5, 22). Cong and Silverstein (23) found a stronger positive wellbeing impact of care provided by daughters-in-law (son's family) than by daughters in rural Anhui, conditional on cultural prescribed expectation. The authors argued that whether the source of care is culturally appropriate or not may be more influential to older parents' wellbeing compared to the support itself. Additionally, some other work investigates the association between gender composition of children and the mortality rate of parents to explore the son preference in patrilineal societies (such as mainland China, Taiwan, and Bangladesh), finding mixed results (24, 25).

## Research Aims and Hypotheses

Due to shrinking family sizes, increasing female empowerment, and weakening traditional patrilineal kinship in China, it has become increasingly common for daughters to provide eldercare to their natal parents (3, 4), and there is growing evidence of a closer relationship between adult daughters and their natal parents (10–12). However, the possible disparity in health outcomes of eldercare provided by daughters and sons has limited attention. This study investigates the difference in the self-reported health of parents who receive eldercare from daughters and sons in China, analyzing the 2013, 2015, and 2018 waves of China Health and Retirement Longitudinal Study (CHARLS). We consider four linked hypotheses.

The first is around the cultural norm and gender difference in socio-economic statuses. In a patrilineal family, sons are expected to fulfill the Confucian ethic of filial piety and undertake the role of primary carers for their old parents (26, 27). In contrast, daughters are considered to be temporary family members. They become a permanent member of the husbands' family after marriage, responsible for taking care of their parents-in-law. In exchange for eldercare, parents invest more in their sons and traditionally would even only leave an inheritance to their sons. Therefore, we expect that sons have a stronger incentive to provide better eldercare (high-quality nutrition, clothing, healthcare services, and living environments). In addition, care from the culturally appropriate source is found to be beneficial for older parents' wellbeing (23). Gender disparities in socio-economic statuses (e.g., income and education) contribute to the difference in the financial capability to provide eldercare between sons and daughters. Older adults' health will be improved with high-quality care, noting that sons are more financially capable and have a stronger incentive to support their parents. Although a number of studies report the decline of these gender-based norms of filial piety and a strengthening of ties between married daughters and their natal parents (4, 6), we still expect the self-reported health of parents who receive care from their sons to be better than those cared for by their daughters (Hypothesis 1).

The second concerns regional disparities which may cause a difference in health outcomes of care provided by sons and daughters. For example, the One-Child Policy was stricter in urban areas. Residents are more likely to have a son in rural areas, as rural couples were allowed to have a second child if the first was a daughter. Compared with urban areas, son preference is much stronger, and economic development is much lower (28). The implementation of nine-year compulsory education in China from 1986 and economic development managed to dramatically close the gender inequality in education and income, especially across urban areas (29, 30). The differences in education between boys and girls mainly exist among poor residents living in rural areas (31). In addition, there are well-known regional inequalities in infrastructure, benefit levels in terms of social welfare, and access to healthcare and social care services where residents living in the rural area are disadvantaged (32, 33). Those inequalities may lead to a higher level of dependence on adult children amongst older parents in rural areas. Thus, we expect that the difference in the outcomes of care provided by sons and daughters is stronger in rural areas (Hypothesis 2).

The third hypothesis draws on the literature around the gendered experience of care. The gender gap in life expectancy from birth (LE) continues at 74.5 for men and 79 for women (34). Combining the gender gap in LE with the gender ideology that women are expected to undertake domestic work, including providing care for families, men are more likely to receive companion and support from their partners (35). In contrast, mothers are more dependent on their children, especially when their partners pass away. Ha et al. (36) supply evidence in favor of the fact that widowed mothers rely on their children for financial and legal advice and instrumental support to a greater extent compared to widowed fathers. Gender norms prescribe men to typically take care of the financial matters of the household (37). Additionally, the literature shows that mothers' wellbeing is more sensitive to the support provided by their adult children (38, 39). Therefore, we expect differences in the outcome of care provided by sons and daughters to be stronger among mothers (Hypothesis 3).

The fourth considers the moderating effect of wealth. Parents with a high level of wealth are able to purchase food and healthcare services and products independent of their children. They can also afford extra care to supplement any insufficient unpaid care provided by their children or spouse. Li et al. (40) analyse data from residents in Shanghai to show that older adults with a higher level of income are more likely to receive formal care. Congruently, Zhu (19) find that older adults with financial independence are less likely to report an unmet need in long-term care. For this reason, wealthier parents' health is less dependent on their children's financial ability and filial piety, and we expect differences in the health outcomes of care provided by daughters and sons to be weaker amongst them (Hypothesis 4).

Our resulting hypotheses are thus

1. The self-reported health of parents who receive care from their sons will be better than those cared for by their daughters.
2. The difference in the health outcomes of care provided by sons and daughters is stronger in rural areas.
3. The difference in the health outcomes of care provided by sons and daughters is expected to be stronger with mothers.
4. The difference in the health outcomes of care provided by sons and daughters is expected to be weaker amongst wealthier parents.

## Methods

### Data and Sample

The nationally representative China Health and Retirement Longitudinal Study (CHARLS) was first conducted in 2011 (41). It collects information on family structure, health, employment, and financial status among those aged 45 and over. Our analysis used waves 2013, 2015, and 2018 of CHARLS with 18,605, 21,095, and 19,816 respondents, respectively. Wave 2011 provides information on whether the respondents received any care from a child's spouse but did not disclose which child the spouse referred to. Without this information, we couldn't tell if the care provided by a child-in-law is from a son's family or a daughter's. For this reason, we exclude Wave 2011 from our analysis.

As there is a fundamental difference in health status between people who need support for daily activities and those who do not, we restrict our sample to those currently receiving instrumental assistances (*n* = 10,936). The health status between parents and their childless peers is also divergent, as people with a lower health status are less likely to have a child (42). We exclude childless adults from our sample, and this process leaves 10,769 observations. Then we cut out the individuals with missing observations of the key variables and drop outliers for household income: the top and bottom 1% of the income. Finally, our sample includes 9,159 observations for 6,594 individuals aged 45 and over who had at least one child and were receiving care during the survey period.

### Dependent Variables

To measure health outcomes, we utilize self-reported health status to create a dummy variable, *SRH_Poor*, following Yiengprugsawan et al. (43): this is equal to one if the respondent reports poor or very poor health and zero if fair, good, very good or excellent health. We refer to it as poor self-reported overall health in the following context if *SRH_Poor* equals 1. For robustness checks, we applied two different measurements for health. The first is a categorical variable for the self-reported health status, *SRH_CA*, consisting of four values: 1 for poor health (poor and very poor health), 2 for fair health, 3 for good health, and 4 for great health (very good and excellent health). Self-reported health has been found to be a reliable physical health measure in multiple studies (44, 45). The second is *Chronic*, capturing the incidence of chronic disease: 1 for no chronic disease, 2 for having chronic disease before, 3 for the onset of new chronic disease since the previous interview.

### Key Independent Variables

CHARLS asked respondents for the information on their primary carers with the following question:

*Who most often help you with dressing, bathing, eating, getting out of bed, using the toilet, controlling for urination and defecation, doing chores, preparing hot meals, shopping, managing money, making phone calls, taking medications?*

**Figure 1:**
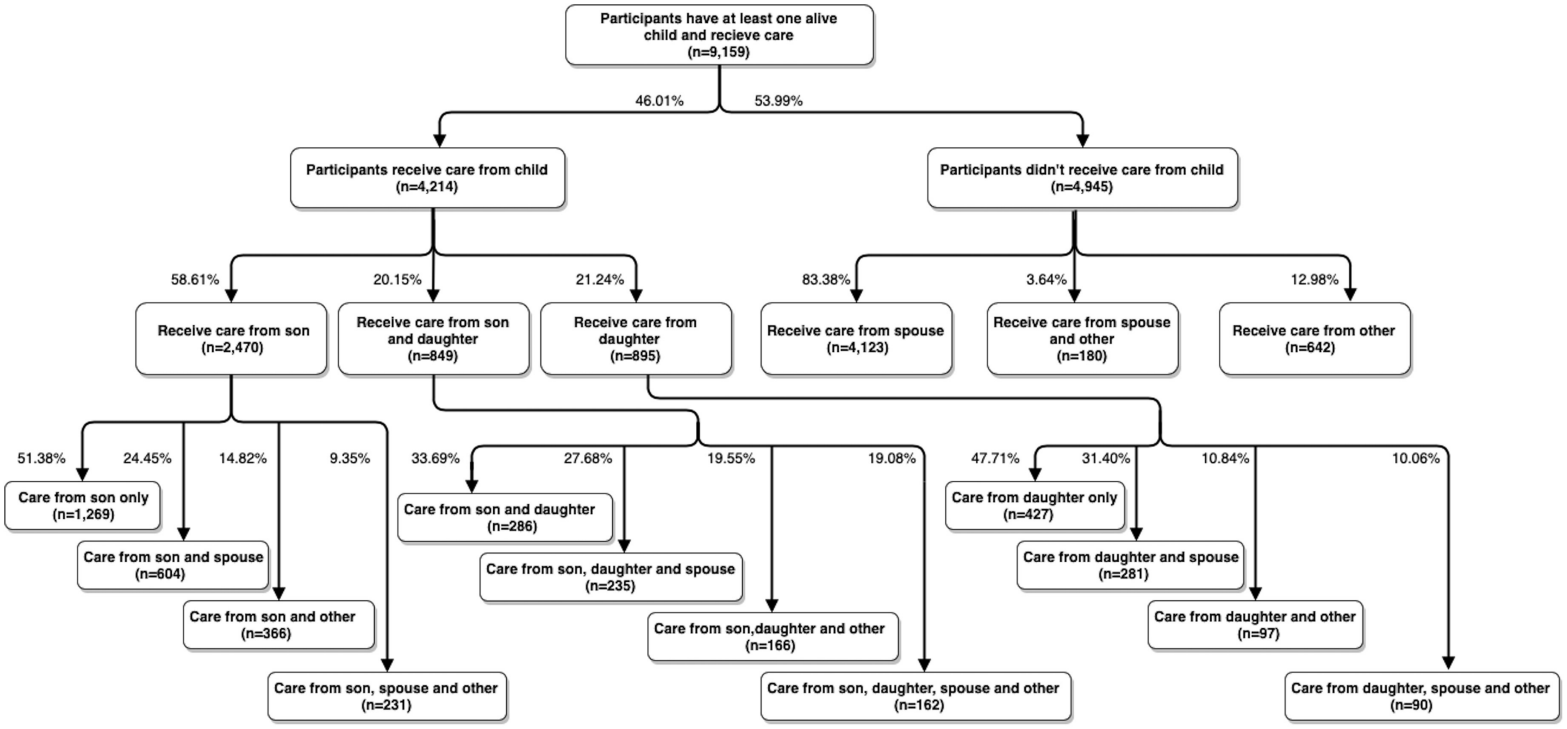
Care structure for people aged 45 and over in China.

Participants were allowed to report up to three persons, and the care structure is displayed in Figure 1. We categorize respondents (*n* = 9,159) into four groups based on the relationship between participants and their primary carers: (a) parents cared for by their son (*n* = 2,470); the participants' primary carers include at least one son (or daughter-in-law) but no daughter (or son-in-law); (b) parents cared for by their daughter (*n* = 895); the participants' primary carers include at least one daughter (or son-in-law) but no son (or daughter-in-law); (c) parents cared for by both their daughter and son (*n* = 849); the participants receive regular care from at least one son (or daughter-in-law) and one daughter (or son-in-law); and (d) parents cared for by others (4,945); the participants obtain regular support from their spouse, siblings, and other relatives or friends rather than their children. We generate a dummy variable for each group: *CaredbySon, CaredbyDaughter, CaredbyBoth* and *CaredbyOther*.

It should be noted that respondents in the above categories a-c may receive care from other people in addition to their children. Among those who receive care from sons but not daughters, 51.38% of them are only cared for by their son(s) (*OnlySonCare, n* = 1,269), and the remaining receive additional support from their spouse or other relatives. For the people who receive care from their daughter(s) but not their son, 47.71% of them are looked after only by their daughter(s) (*OnlyDaughterCare, n* = 427). As a cursory robustness check, we restrict our sample to those cared for only by their son(s) or only by their daughter(s).

### Baseline Model

To investigate whether parents who are receiving care from their son(s) have a better self-reported health status than those who obtain care from their daughter(s), we estimate the following model:


(1) $$\begin{array}{l} SRH\_Poor_{i,t}\;=\beta_{0}+\beta_{1}CaredbySon_{i,t}\\ \;+\;\beta_{2}CaredbyDaughter_{i,t}+\beta_{3}CaredbyBoth_{i,t}\\ \;+\;\beta_{4}*Controls_{i,t}+u_{i}+u_{t}+\epsilon_{i,t} \end{array}$$

*SRH*_*Poor*_(*i, t*)_ is the self-reported health status for individual *i* in year *t*. *CaredbySon*__*i*_, t_, *CaredbyDaughter*_*i, t*_, and *CaredbyBoth*_*i, t*_ are the dummy variables indicating the care structure as described above. The reference group in the model is those who didn't receive any care from their children (*CaredbyOhter*_*i, t*_ = 1). If the care provided by a son outperforms that by a daughter (Hypothesis 1), we would expect β_1_ to be significant and smaller than β_2_. *Controls*_*i, t*_ are other factors identified in the literature as essential determinants of health status: gender, age, marital status, education attainment, financial status, employment status, and family structure (17). The definitions of all variables are listed in Appendix (Supplementary Table A1). *u*_*i*_ *and u*_*t*_ represent the individual-specific time-invariant effects and the business cycle effects, respectively. ϵ_*i, t*_ is an idiosyncratic error term.

Restricting our sample to those who only receive care from sons or daughters, we estimate the following equation:


(2) $$\begin{array}{l} SRH\_Poor_{i,t}\;=\beta_{0}+\beta_{1}OnlySonCare_{i,t}\\ \;+\beta_{2}\;*\;Controls_{i,t}+u_{i}+u_{t}+\epsilon_{i,t} \end{array}$$

*OnlySonCare*_*i, t*_ is equal to one if individual *i* is only looked after by their son(s) in year *t* and zero otherwise. In this model, the reference group is parents who only receive care from their daughter(s). If Hypothesis 1 cannot be rejected, the random-effects logistic estimator, β_1_(odds ratio), would be significantly smaller than 1. To examine Hypotheses 2–4, we estimate Equations 1 and 2 with subsamples of urban and rural, mothers and fathers and participants with a high and lower wealth level.

## Results

### Descriptive Statistics

Table 1 shows our sample characteristics. Among those who receive care and have at least one child (*N* = 9,195), 36.47% of them are male (*N* = 3,340), and 31.63% live in urban areas. This group's average age is about 66, 77.55% of the whole sample are married, and 18.47% are widowed. Among those looked after by their daughter but not by their son, 44.02% of them do not have an alive son. Compared with parents who receive care from a son but not their daughter, they are more likely to be married (67.82% vs. 58.54%), complete primary education (33.97% vs. 25.02%), live in the urban areas (39.66% vs. 28.99%) and engage in non-agriculture working (6.70% vs. 4.78%). They also tend to be younger (mean age 65.63 vs. 68.60) and have a higher household income (RMB 26,181 vs. RMB 22,421) and are less likely to be widowed (27.26% vs. 36.40%). For those whose primary carers include their son but not their daughter, 26.60% of them do not have a daughter.

**Table 1:** Sample characteristics.

	**Full sample**	**Care providers**			
		**Son**	**Daughter^a^**	**Both**	**Other**
	***N =* 9,195**	***N =* 2,470**	***N =* 895**	***N =* 849**	***N =* 4,945**
	***M* (SD)**	***M* (SD)**	***M* (SD)**	***M* (SD)**	***M* (SD)**
Male	0.3647 (0.4814)	0.2980 (0.4575)	0.2793 (0.4489)	0.3004 (0.4587)	0.4245 (0.4943)
Age	65.9388 (10.4904)	68.5951 (10.7649)	65.6257^***^ (11.1680)	67.6231 (11.3744)	64.3794 (9.7350)
Married	0.7755 (0.4173)	0.5854 (0.4927)	0.6782^***^ (0.4674)	0.6337 (0.4821)	0.9124 (0.2827)
Widowed	0.1847 (0.3881)	0.3640 (0.4812)	0.2726^***^ (0.4456)	0.2980 (0.4576)	0.0599 (0.2372)
Primary education	0.335 (0.4721)	0.2502 (0.4332)	0.3397^***^ (0.4739)	0.3039 (0.4602)	0.3824 (0.4860)
Urban	0.3163 (0.4651)	0.2899 (0.4538)	0.3966^***^ (0.4895)	0.3663 (0.4821)	0.3064 (0.4610)
Real household income	23500.89 (35196.50)	22421.43 (33737.13)	26180.88^**^ (37558.93)	23357.36 (34445.84)	23579.66 (35577.79)
Logarithm of real household income	8.5679 (2.6505)	8.5026 (2.6507)	8.5130 (2.9540)	8.4619 (2.8132)	8.6286 (2.5613)
Real household wealth	23427.43 (53558.87)	21806.02 (50209.44)	24811.71 (57142.19)	25821.83 (62545.43)	23559.21 (52864.62)
Logarithm of real household wealth	8.7928 (1.7076)	8.6800 (1.7652)	8.8360^*^ (1.7252)	8.7165 (1.8362)	8.8497 (1.6529)
Farming	0.3924 (0.4883)	0.3506 (0.4773)	0.3263 (0.4691)	0.3475 (0.4764)	0.4330 (0.4955)
Non-agriculture working	0.0701 (0.2553)	0.0478 (0.2133)	0.0670^*^ (0.2502)	0.0766 (0.2660)	0.0807 (0.2724)
Number of sons	1.6747 (1.0952)	2.0688 (1.0968)	0.9140^***^ (1.0408)	1.8339 (0.9625)	1.5883 (1.0354)
Number of daughters	1.5517 (1.2039)	1.4628 (1.3117)	2.1654^***^ (1.1200)	1.8257 (0.9883)	1.4380 (1.1546)
No son	0.0976 (0.2968)	0.0008 (0.0284)	0.4402^***^ (0.4967)	0.0000 (0.0000)	0.1007 (0.3010)
No daughter	0.1783 (0.3828)	0.2660 (0.4419)	0.0000^***^ (0.0000)	0.0012 (0.0343)	0.1972 (0.3979)

a *We have conducted mean-comparison tests between the care provider groups of Son and Daughter*.

* *p < 0.05*,

** *p < 0.01*,

*** *p < 0.001*.

Table 2 displays the health status of participants by care structure, indicating a better health outcome of care provided by their son. A larger proportion of parents whose primary carers include their daughter(s) but not their son(s) report poor health (57.99% vs. 50.93%) and have had a new onset of chronic disease (42.37% vs. 35.81%) since the previous interview, compared with those whose primary carers include their son(s) but not their daughter(s).

**Table 2:** Health status by care providers.

	**Full sample**	**Care providers**			
		**Son**	**Daughter^a^**	**Both**	**Other**
	***N =* 9,195**	***N =* 2,470**	***N =* 895**	***N =* 849**	***N =* 4,945**
	***M* (SD)**	***M* (SD)**	***M* (SD)**	***M* (SD)**	***M* (SD)**
**Self-reported health**
1 poor	0.5275 (0.4993)	0.5093 (0.5000)	0.5799^***^ (0.4939)	0.5253 (0.4997)	0.5274 (0.4993)
2 fair	0.3643 (0.4813)	0.3664 (0.4819)	0.3240^*^ (0.4683)	0.3804 (0.4858)	0.3678 (0.4823)
3 good	0.0657 (0.2478)	0.0826 (0.2753)	0.0592^*^ (0.2362)	0.0554 (0.2288)	0.0603 (0.2380)
4 great	0.0425 (0.2017)	0.0417 (0.1999)	0.0369 (0.1886)	0.0389 (0.1934)	0.0445 (0.2062)
**Chronic disease**
1 no chronic disease	0.2668 (0.4423)	0.2569 (0.4370)	0.2450 (0.4303)	0.2534 (0.4352)	0.2778 (0.4480)
2 onset before	0.3518 (0.4776)	0.3849 (0.4867)	0.3314^**^ (0.4710)	0.332 (0.4713)	0.3424 (0.4746)
3 new onset	0.3814 (0.4858)	0.3581 (0.4796)	0.4237^***^ (0.4944)	0.4145 (0.4929)	0.3797 (0.4854)

a *We have conducted mean-comparison tests between the care provider groups of Son and Daughter*.

* *p < 0.05*,

** *p < 0.01*,

*** *p < 0.001*.

### Self-Reported Health Status and Care Structure

The random-effects logistic regression estimates for Equations 1 and 2 are reported in Table 3. In comparison to those who receive care from others rather than their own children, Column 1 shows that the odds ratio of having poor overall health vs. not poor health is similar amongst parents who receive care from their son (OR 0.985, 95% CI 0.839–1.158) and higher among parents who receive care from their daughter (1.453, 1.152–1.833). We have conducted an *F*-test to compare the self-reported health of parents who receive care from their sons with their daughters, and the result is reported at the end of the table. It confirms that the odds ratio of having poor health is statistically and significantly higher among parents who are cared for by their daughter(s) than those cared for by their son(s) (*p* = 0.002). The analysis shown in the second column restricts the sample to parents who only receive care from their sons or daughters. The odds ratio of having poor health for parents cared for only by their son(s) is 0.636 (0.439–0.919) times that of parents looked after only by their daughter(s).

**Table 3:** Random-effects logistic regression models for self-reported health and care provided by son and daughter.

	**(1)**	**(2)**
	**Model 1**	**Model 2**
Male	1.0642 (0.9122–1.2416)	0.9145 (0.6471–1.2924)
Age	0.9977 (0.9894–1.0061)	0.9793^*^ (0.9628–0.9962)
**Marital status (ref. single or divorced)**		
Married	1.1813 (0.8479–1.6458)	0.9095 (0.5081–1.6280)
Widowed	0.9430 (0.6514–1.3650)	1.0305 (0.5612–1.8923)
Primary education	1.3871^***^ (1.1814–1.6287)	1.0703 (0.7374–1.5535)
Household income	0.9663^**^ (0.9427–0.9906)	0.9520 (0.9058–1.0005)
Working	0.4606^***^ (0.3556–0.5967)	0.9555 (0.4365–2.0917)
Number of sons	1.0472 (0.9737–1.1263)	1.1532 (0.9971–1.3338)
Number of daughters	1.0525 (0.9881–1.1211)	1.1247 (0.9953–1.2710)
**Care structure (ref: CaredbyOther)**		
CaredbyDaughter	1.4528^**^ (1.1516–1.8326)	
CaredbySon	0.9854 (0.8389–1.1575)	
CaredbyBoth	1.0910 (0.8749–1.3605)	
**Care structure (ref: OnlyDaughterCare)**		
OnlySonCare		0.6356^*^ (0.4394–0.9192)
Urban	0.7911^**^ (0.6741–0.9284)	0.7313 (0.5222–1.0239)
Constant	0.9466 (0.4515–1.9844)	3.6458 (0.8219–16.1723)
Observations	9,159	1,694
Number of individuals	6,594	1,524
Wald chi2	162.88	45.25
AIC	12232	2323
*p*-value	0.0024	

*We use a random-effects logistic model, and the odds ratios (0Rs) are reported. The 95% confidence intervals are reported in parentheses. Year and province dummies are included in all models, but their coefficients are not reported for brevity. The P-value of F-test for the equality of coefficients on the ‘CarebyDaughter' and ‘CarebySon' is reported at the end of the table. See Appendix for the complete definitions of all variables*.

* *p < 0.05*,

** *p < 0.01*,

*** *p < 0.001*.

### The Difference in Outcomes of Care Provided by Sons and Daughters by Region: Rural vs. Urban

We differentiate parents based on the regions in which they are living and compare the difference in the health outcome of care provided by daughters and sons between rural and urban areas, finding that this disparity mainly exists in rural areas (Table 4). In urban areas, there is no difference in the odds ratio of having poor health among parents who receive care from their children and those cared for by others. However, the parents cared for by their daughter(s) report a lower level of health status in rural areas (Column 2, OR 1.676, 95% CI 1.247–2.253). The difference in the odds ratio of having poor health among parents who receive care from daughters and from sons is statistically significant in rural areas (*p* = 0.003) but not in urban areas (*p* = 0.195). Consistently, Column 3 shows no significant difference in the health status of parents who receive care only from their daughter and those only from their son in urban areas (Column 3, OR = 0.877, 95% CI 0.454–1.693). The odds ratio of having poor health for parents cared for only by their son(s) is 0.620 (0.395–0.972) times that of people who receive care only from their daughter(s) in rural areas (Column 4).

**Table 4:** Radom-effects logistic regression models for self-reported health and care provided by son and daughter: differentiating by region.

	**Model 1**		**Model 2**	
	**(1)**	**(2)**	**(3)**	**(4)**
	**Urban**	**Rural**	**Urban**	**Rural**
Male	0.9736 (0.7442–1.2736)	1.1021 (0.9125–1.3310)	1.1129 (0.5713–2.1680)	0.8912 (0.5935–1.3382)
Age	0.9875 (0.9730–1.0022)	1.0017 (0.9914–1.0122)	0.9805 (0.9483–1.0138)	0.9801^*^ (0.9606–1.0000)
**Marital status (ref. single or divorced)**
Married	0.9650 (0.5212–1.7867)	1.2643 (0.8540–1.8718)	0.6428 (0.2166–1.9080)	1.0302 (0.5090–2.0849)
Widowed	0.7589 (0.3824–1.5062)	1.0057 (0.6488–1.5590)	0.6748 (0.2162–2.1062)	1.1679 (0.5596–2.4372)
Primary education	1.3133^*^ (1.0033–1.7190)	1.3889^**^ (1.1354–1.6991)	0.8481 (0.4486–1.6035)	1.1169 (0.6965–1.7912)
Household income	0.9828 (0.9434–1.0237)	0.9580^**^ (0.9284–0.9885)	0.9836 (0.9075–1.0661)	0.9386 (0.8807–1.0002)
Working	0.3737^***^ (0.2411–0.5791)	0.5133^***^ (0.3706–0.7109)	0.8115 (0.2134–3.0865)	1.1045 (0.3957–3.0832)
Number of sons	0.9924 (0.8710–1.1307)	1.0729 (0.9815–1.1729)	0.9075 (0.6727–1.2243)	1.2131^*^ (1.0184–1.4450)
Number of daughters	1.0101 (0.9022–1.1310)	1.0764 (0.9968–1.1624)	1.0357 (0.8207–1.3069)	1.1724^*^ (1.0106–1.3601)
**Care structure (ref: CaredbyOther)**
CaredbyDaughter	1.1510 (0.7851–1.6872)	1.6762^***^ (1.2473–2.2526)		
CaredbySon	0.8680 (0.6419–1.1738)	1.0411 (0.8594–1.2611)		
CaredbyBoth	1.3623 (0.9351–1.9847)	0.9806 (0.7455–1.2897)		
**Care structure (ref: OnlyDaughterCare)**
OnlySonCare			0.8770 (0.4543–1.6931)	0.6196^*^ (0.3950–0.9721)
Constant	2.6670 (0.6405–11.1048)	0.5837 (0.2391–1.4249)	1.5492 (0.0626–38.3305)	3.0146 (0.5304–17.1337)
Observations	2,897	6,262	557	1,134
Number of individuals	2,152	4,442	502	1,019
Wald chi2	71.89	113.42	18.27	33.06
AIC	3905.247	8358.679	790.379	1563.392
*p*-value	0.1952	0.0030		

*We use a random-effects logistic model, and the odds ratios (0Rs) are reported. The 95% confidence intervals are reported in parentheses. Year and province dummies are included in all models, but their coefficients are not reported for brevity. The P-values of F-tests for the equality of coefficients on the ‘CarebyDaughter' and ‘CarebySon' are reported at the end of the table*.

* *p < 0.05*,

** *p < 0.01*,

*** *p < 0.001*.

### The Difference in Outcomes of Care Provided by Sons and Daughters by Care Recipients: Mother vs. Father

Table 5 displays the results for mothers and fathers separately. Among fathers, there is no significant difference in the odds ratio of having poor health between those who receive care from children and those cared for by others (Column 1). Mothers looked after by others rather than children have a similar health status with those cared for by their son(s) (Column 2: OR = 0.960, 95% CI 0.782−1.178), while women cared for by their daughters(s) report a lower level of health status (Column 2: OR = 1.494, 95% CI 1.122−1.988). Focusing on the sample of people cared for only by sons or only by daughters, there is no difference in the health statuses between fathers who are looked after only by sons and by daughters (Column 3: OR = 0.502, 95% CI 0.242−1.042). However, the odds ratio for mothers cared for only by their son(s) is 0.644 (0.420−0.989) times that of those cared for only by their daughter(s).

**Table 5:** Radom-effects logistic regression models for self-reported health and care provided by son and daughter: differentiating by gender.

	**Model 1**		**Model 2**	
	**(1)**	**(2)**	**(3)**	**(4)**
	**Male**	**Female**	**Male**	**Female**
Age	0.9966 (0.9835–1.0100)	0.9981 (0.9873–1.0090)	0.9698 (0.9375–1.0032)	0.9829 (0.9640–1.0022)
**Marital status (ref. single or divorced)**
Married	0.8942 (0.5023–1.5917)	1.2961 (0.8604–1.9523)	0.4906 (0.1784–1.3488)	1.2851 (0.6358–2.5977)
Widowed	0.6811 (0.3506–1.3230)	1.0514 (0.6675–1.6559)	0.5987 (0.2111–1.6976)	1.3853 (0.6667–2.8782)
Primary education	1.5337^***^ (1.2205–1.9274)	1.2612^*^ (1.0059–1.5812)	1.3949 (0.7931–2.4534)	0.9074 (0.5632–1.4620)
Household income	0.9456^**^ (0.9077–0.9851)	0.9817 (0.9511–1.0133)	0.9201 (0.8302–1.0199)	0.9731 (0.9195–1.0297)
Working	0.3480^***^ (0.2414–0.5019)	0.5621^**^ (0.3901–0.8099)	0.5541 (0.1674–1.8339)	1.3191 (0.4957–3.5102)
Number of sons	0.9770 (0.8688–1.0986)	1.0895 (0.9923–1.1963)	1.0363 (0.8113–1.3237)	1.2170^*^ (1.0213–1.4502)
Number of daughters	1.0467 (0.9499–1.1533)	1.0541 (0.9698–1.1457)	1.3065^*^ (1.0239–1.6671)	1.0300 (0.8903–1.1916)
**Care structure (ref: CaredbyOther)**
CaredbyDaughter	1.3853 (0.9162–2.0946)	1.4935^**^ (1.1221–1.9880)		
CaredbySon	1.0032 (0.7693–1.3081)	0.9600 (0.7824–1.1779)		
CaredbyBoth	1.4225 (0.9668–2.0929)	0.9719 (0.7388–1.2787)		
**Care structure (ref: OnlyDaughterCare)**
OnlySonCare			0.5023 (0.2422–1.0417)	0.6441^*^ (0.4196–0.9887)
Urban	0.7756^*^ (0.6049–0.9945)	0.8044^*^ (0.6522–0.9921)	0.9099 (0.5099–1.6237)	0.6812 (0.4555–1.0186)
Constant	1.551 6 (0.4645–5.1825)	0.7902 (0.3036–2.0571)	11.9358 (0.4283–332.6507)	2.1821 (0.3962–12.0193)
Observations	3,340	5,819	423	1,270
Number of individuals	2,499	4,105	402	1,121
Wald chi2	98.45	97.28	11.91	37.55
AIC	4480.623	7782.467	610.048	1748.256
*p*-value	0.1593	0.0048		

*We use a random-effects logistic model, and the odds ratios (0Rs) are reported. The 95% confidence intervals are reported in parentheses. Year and province dummies are included in all models, but their coefficients are not reported for brevity. The P-values of F-tests for the equality of coefficients on the ‘CarebyDaughter' and ‘CarebySon' are reported at the end of the table*.

* *p < 0.05*,

** *p < 0.01*,

*** *p < 0.001*.

### The Difference in Outcomes of Care Provided by Sons and Daughters by Household Wealth

To investigate the moderating effect of wealth on the difference in health outcomes of care provided by sons and daughters, we differentiate participants based on their household wealth. We define participants whose wealth is in the top 25% of the distribution as wealthy parents, and the results are reported in Table 6. Care structure is not associated with wealthy parents' health: there is no difference in the odds ratio of having poor health among wealthy parents who receive care from their children and others (Column 1). Consistently, Column 3 shows that wealthy parents cared for only by their daughter have a similar health status with parents looked after solely by their son (0.290, 0.035–2.413). In contrast, less wealthy parents report a higher level of health status if they only receive care from their son (Column 4, OR = 0.681, 95% CI 0.471–0.985).

**Table 6:** Radom-effects logistic regression models for self-reported health and care provided by son and daughter: differentiating by wealth.

	**Model 1**		**Model 2**	
	**(1)**	**(2)**	**(3)**	**(4)**
	**Wealthy**	**Less wealthy**	**Wealthy**	**Less wealthy**
Male	1.0297 (0.7402–1.4325)	1.0446 (0.8802–1.2398)	2.5835 (0.4520–14.7680)	0.8210 (0.5722–1.1779)
Age	1.0106 (0.9938–1.0276)	0.9927 (0.9831–1.0024)	0.9596 (0.8861–1.0392)	0.9751^**^ (0.9575–0.9930)
**Marital status (ref. single or divorced)**
Married	0.6497 (0.2788–1.5143)	1.3374 (0.9326–1.9178)	0.4558 (0.0229–9.0787)	0.9341 (0.5229–1.6686)
Widowed	0.5109 (0.1998–1.3064)	1.1100 (0.7417–1.6610)	0.2689 (0.0100–7.1970)	1.1930 (0.6535–2.1778)
Primary education	1.4439^*^ (1.0147–2.0546)	1.4311^***^ (1.1957–1.7129)	0.3025 (0.0413–2.2157)	1.2264 (0.8394–1.7919)
Household income	0.9424^*^ (0.8932–0.9944)	0.9792 (0.9516–1.0076)	0.8215 (0.6312–1.0693)	0.9800 (0.9295–1.0331)
Working	0.3539^***^ (0.2010–0.6230)	0.5211^***^ (0.3872–0.7012)	0.0508 (0.0015–1.6731)	1.3173 (0.5745–3.0202)
Number of sons	0.9495 (0.8064–1.1180)	1.0754 (0.9920–1.1658)	1.0374 (0.5380–2.0003)	1.2274^**^ (1.0522–1.4318)
Number of daughters	0.9405 (0.8203–1.0782)	1.0796^*^ (1.0055–1.1591)	1.0907 (0.6192–1.9213)	1.1526^*^ (1.0148–1.3092)
**Care structure (ref: CaredbyOther)**
CaredbyDaughter	1.3965 (0.8464–2.3043)	1.4274^**^ (1.1012–1.8504)		
CaredbySon	0.9275 (0.6345–1.3557)	1.0188 (0.8517–1.2188)		
CaredbyBoth	0.9345 (0.5545–1.5749)	1.1653 (0.9080–1.4953)		
**Care structure (ref: OnlyDaughterCare)**
OnlySonCare			0.2897 (0.0348–2.4128)	0.6809^*^ (0.4705–0.9853)
Urban	0.8907 (0.6367–1.2461)	0.8069^*^ (0.6737–0.9663)	0.4626 (0.1000–2.1403)	0.8170 (0.5843–1.1425)
Constant	0.5996 (0.1149–3.1308)	1.2709 (0.5550–2.9105)	384.7024 (0.2765–535,247.4565)	5.9655^*^ (1.2707–28.0061)
Observations	1,816	7,343	416	1,278
Number of individuals	1,626	5,400	403	1,168
Wald chi2	41.76	122.35	4.96	35.77
AIC	2487.810	9811.611	572.347	1769.196
*p*-value	0.1348	0.0189		

*We use a random-effects logistic model, and the odds ratios (0Rs) are reported. The 95% confidence intervals are reported in parentheses. Year and province dummies are included in all models, but their coefficients are not reported for brevity. The P-values of F-tests for the equality of coefficients on the ‘CarebyDaughter' and ‘CarebySon' are reported at the end of the table*.

* *p < 0.05*,

** *p < 0.01*,

*** *p < 0.001*.

### Sensitivity Analysis

In addition to a dummy variable indicating overall poor health, we also use a category variable for self-reported health, and the incidences of chronic disease as additional measurements for health status. The results are displayed in the Appendix (Supplementary Tables A2–A6). The results consistently show that parents cared for by their son(s) have a higher odds ratio of having great health (*SRH_CA* = 4) vs. the combined good (*SRH_CA* = 3), fair (*SRH_CA* = 2) and poor (*SRH_CA* = 1) health and are less likely to have a new onset of chronic disease than those parents looked after by their daughters. The difference in health outcomes of care given by daughters and sons exists in rural areas for mothers and less wealthy parents.

## Discussion

The traditional eldercare system in China, where family members (and especially sons) are expected to look after their older parents, is challenged by low fertility rates and urbanization progress (46). In past decades, there has been an increasing number of parents receiving care from their daughters, triggered by the reduced availability of sons, women's empowerment, and modernization (2–4). We compared the health status of the parents who receive care from their daughters and their sons with nationally representative data.

Our results show that parents who receive eldercare from their son(s) report a higher level of self-reported health status than those cared for by their daughter(s), consistent with our first hypothesis. A pragmatic explanation might be the gender difference in the motivation and financial capability of providing eldercare. In a patrilineal society such as China, traditionally, all the family property will be divided solely among sons, and parents invest a larger amount in their son than their daughter. Sons are also expected to take care of their parents following the Confucian ethic of filial piety (26, 27). Therefore, sons have a stronger motivation to provide high-quality eldercare to their parents than daughters.

Along with social and economic changes, cultural necessities mandate the role that daughters typically play in the provision of eldercare for their natal parents (15, 17). In terms of a growing labor force participation, economic development increased women's empowerment and their subsequent household decision-making and financial capacity (47), which enable daughters to support their natal parents. However, the stratified gender differences in the social-economic status, income, and education, implies that sons can provide their parents with better food and healthcare service and products and more comfortable and convenient living arrangements, which improves their parents' health.

An alternative explanation concerns the influence of gendered expectations. In particular, parents with a strong prior son preference may report better health if they receive care from their son. We attempted to control for this factor through the inclusion of robustness tests where the diagnoses of new chronic diseases are applied as indicators of health outcomes. However, we also acknowledge that there may be a psycho-social element to these more objective variables. Older parents feeling less secure with daughter-care may experience stress leading to chronic disease, for example. Or they may perceive that their daughters are not able to physically support them and thus neglect to ask for assistance in moving around, leading to a worse wellbeing when daughters are the only carers.

The gender difference in the outcome of care provided by sons and daughters is greater in rural areas: our 2nd hypothesis was supported by the data. Indeed, the difference in the self-reported health outcome of care provided by sons and daughters was mainly exhibited by those living in rural areas. The One-Child Policy in China was more effective in urban areas, reducing both the availability of sons and also cultural son preference (48). In addition, the higher levels of education received by girls in urban settings have enabled them to increase their economic employment and thus the ability to provide care for their older parents (49). Higher education for both men and women also changes cultural norms and preferences and a greater acceptance by both of the necessity of daughters to provide care to their natal parents in the absence of sons (50). All of these factors enhance the daughter's incentive and capability to provide good eldercare to their parents in urban areas. However, many of these factors are not yet present in rural areas, so traditional cultural norms of behavior and expectations still exist.

Our third hypothesis was that the gender difference in the outcome of care provided by sons and daughters was expected to be stronger among mothers. This is also supported by the data as only mothers and not fathers experience this gender externality. Men are more likely to receive support and care from their spouse (51). The assistance given to men by female spouses will alleviate their dependency on their children, even in the case that the spouse is not the primary carer. Finally, we found that the differences in the health outcomes of care provided by daughters and sons were weaker amongst wealthy parents, Hypothesis 4. Wealthy parents could afford extra care to supplement the insufficient support offered by their children or other family members. They are also able to pay for high-quality healthcare services by themselves even if their children cannot provide it for them. A lower dependency on children contributes to a weaker gender difference in the health outcome of children's care amongst fathers and wealthy people.

Our results are inconsistent with some recent empirical studies which show daughter advantage in providing instrumental and emotional care (17–19), echoing Cong and Silverstein (23) and Liu and Harper (5). These differences are contributed to by divergent samples and various measurements for primary carer and outcomes. Three of the empirical studies analyzed the China Longitudinal Healthy Longevity Survey (CLHLS). Zhu (19) examined the older parents aged 80 and over with the 2005–2011 waves of CLHLS, and Zeng et al. (17, 18) focused on the group aged 65 and over with 2002–2008 waves of CLHLS. It is very likely that the Family Plan Policy hadn't impacted those samples, and the average number of living children was 3.4 and 3.2 in urban and rural areas in Zhu (19) and 3.6 and 3.8 in Zeng et al. (18). The dataset asks the participant to report one primary carer, even though it is prevalent for multiple children to share caring responsibilities (52).

CHARLS, conversely, asked participants to report up to three primary carers if they receive regular care from multiple sources. Figure 1 demonstrates that daughters usually care for their parents with their siblings. Of the 4,214 parents who receive care from their children, only 21.24% are cared for by daughters but not sons, 58.61% are looked after by their sons but not daughters, and the remaining are supported by both. Among those whose primary carers include a daughter but not a son, 44.02% of them do not have an alive son. Based on their measurement of the carer, parents with a daughter as the primary carer are very likely to receive support from their sons as well. Their method largely underestimates the contributions made by the son.

Our study suffers from the following limitations. The primary measurement of health outcome in the study is self-reported health status, which could involve measurement error and be influenced by the respondent's mood or other factors during the interview. Future research could use other better measurements of health, such as biomarkers. There may also exist endogeneity between health status and care structure (e.g., preference of the primary carers). Therefore, our results cannot be interpreted as causal. However, we can use the association between health status and care structure to identify the people who are suffering from health inequality. When the data becomes available, it would be interesting to investigate the main mechanism behind the gender differences in health outcomes of care provided by children: gender difference in the motivation of providing care or the gender disparity in financial capability or gender expectations; and its heterogeneity among regions and cohorts.

Despite these limitations, our study contributes to the understanding of intergenerational support and health inequalities associated with the care system in China, with the following implications. As China modernizes, higher education for females will become more widespread, and women become more economically independent; their structural ability to provide high-quality care and the cultural acceptance of this care will increase. This is already the case in most urban areas of China, where higher education for both men and women has already changed cultural norms and preferences around daughter care. As these drivers spread to rural areas, so we would expect to see a great ability and acceptability of daughter care within all families. In order to support this transition in rural areas, our finding suggests that reducing dependency on children via seeking supplementary or substitution of care given by children may reduce this inequality.

Given the rapid aging speed in China, the accessibility of good quality of care is of great concern (53, 54). It becomes challenging for the only child to be the primary carer for their parents and parents-in-law (55). The current (formal) long-term care system in China is characterized by a growing provision of residential care with a decreasing bed occupancy rate, a low availability of home and community-based services and a lack of quality regulations and funding (33). One policy recommendation of our study is for governments to enhance the infrastructures for care services and promote the innovations of care products, for instance, care homes, nursing homes, and day-care centers, creating more choices for older parents to arrange their care. Our findings with respect to regional inequality encourage further policy attention and resource allocation, especially in rural areas.

Guiding older adults to arrange their care in order to help them understand the advantages of outsourcing care services is also important. It is still common in China for older adults to be reluctant to live in nursing homes, as they view it as stigma against the cultural norm (55). Actions are required to enhance the social acceptability of outsourced care products and services and encourage older adults to voluntarily seek the substitution of the care provided by their children. To increase older adults' independence, it remains crucial to improve their financial capability, which could be done through a reliable and comprehensive pension system.

Responding to population aging with a low fertility rate in China, the central government has gradually relaxed the Family Plan Policy since the 6^th^ national population census in 2010 (56). At the time of writing, each couple is allowed to have up to three children (from May 2021 to date). It has already been argued that relaxing the Family Plan Policy alone has done little to improve the fertility rate, which appears due to a variety of complex interactions including new norms of child bearing, the high costs of rearing a child and the responsibility of eldercare (57). Therefore, developing the long-term care system may alleviate the family burden of adults of reproductive age and, in return, encourage them to have more children. However, it is worth noting that if the measure boosts fertility in the short term, the demand for childcare will further reduce the availability of family care for older adults within the currently underdeveloped long-term care and childcare system.

Collaboration across governments, the third sector, public bodies and the broader general public is needed to reduce health inequality among older adults as China transitions through economic development.

## Data Availability Statement

The original CHARLS survey data is publicly available (http://charls.pku.edu.cn/index/en.html).

## Author Contributions

SH planned the study, supervised the data analysis, and co-wrote the paper. YZ designed and undertook the statistical analyses and co-wrote the paper. Both authors contributed to the article and approved the submitted version.

## Funding

This work was supported by the Clore Duffield Foundation (Grant Number: R74553).

## Conflict of Interest

The authors declare that the research was conducted in the absence of any commercial or financial relationships that could be construed as a potential conflict of interest.

## Publisher's Note

All claims expressed in this article are solely those of the authors and do not necessarily represent those of their affiliated organizations, or those of the publisher, the editors and the reviewers. Any product that may be evaluated in this article, or claim that may be made by its manufacturer, is not guaranteed or endorsed by the publisher.
