# Supplementary material for: Son or Daughter Care in Relation to Self-Reported Health Outcomes for Older Adults in China
Source: Front Public Health. 2022 Jan 18;9:793873. doi: 10.3389/fpubh.2021.793873 (PMC8805801; doi:10.3389/fpubh.2021.793873)
Supplement: Supplementary file 1 [file Data_Sheet_1.pdf]

Supplementary material

Table A1: Definition of variables

Variable	Definition
**Male**	Equal to 1 if the respondent is male, 0 otherwise
**Age**	Respondent's age
**Married**	Equal to 1 if the respondent is married, 0 otherwise
**Widowed**	Equal to 1 if the respondent is widowed, 0 otherwise
**Primary Education**	Equal to 1 if the respondent finished primary education, 0 otherwise
**Real Household Income**	Annual household income at 2018 prices
**Logarithm of real household income**	Logarithm of annual household income at 2018 prices
**Real Household wealth**	Non-housing household wealth at 2018 prices
**Logarithm of real household wealth**	Non-housing household wealth at 2018 prices
**Working**	Equal to 1 if the respondent does non-agriculture work, 0 otherwise
**Number of Sons**	The number of respondents' sons
**Number of Daughters**	The number of respondents' daughters
**CaredbyDaughter**	Equal to 1 if the respondent's daughter is the main carer but not son, 0 otherwise
**CaredbySon**	Equal to 1 if the respondent's son is the main carer but not daughter, 0 otherwise
**CaredbyBoth**	Equal to 1 if the respondents are cared by both daughter and son, 0 otherwise
**CaredbyOther**	Equal to 1 if the respondent is mainly cared for by others rather than children, 0 otherwise
**OnlyDaughterCare**	Equal to 1 if the respondent's primary carer is daughter only, 0 otherwise
**OnlySonCare**	Equal to 1 if the respondent's primary carer is son only, 0 otherwise
**SRH_Poor**	Equal to 1 if the respondent reports very poor or poor health, 0 otherwise
**SRH_CA**	Self-reported health: 1 poor; 2 fair; 3 good; 4 excellent
**Chronic**	Category of chronic disease: 1 No chronic disease; 2 Onset before; and 3 Onset new

Table A2: Ordinal logistic regression models for self-reported health and care provided by son and daughter

	(1)<br>Model 1	(2)<br>Model 2
**Male**	1.014	1.104
	(0.925 - 1.110)	(0.877 - 1.388)
**Age**	1.004	1.016**
	(0.998 - 1.009)	(1.005 - 1.027)
**Marital status (ref. single or divorced)**		
**Married**	0.908	0.937
	(0.737 - 1.119)	(0.631 - 1.392)
**Widowed**	1.091	0.902
	(0.865 - 1.376)	(0.597 - 1.364)
**Primary Education**	0.764***	0.900
	(0.694 - 0.841)	(0.705 - 1.147)
**Household Income**	1.021*	1.032
	(1.004 - 1.037)	(0.997 - 1.068)
**Working**	1.704***	1.148
	(1.453 - 1.999)	(0.689 - 1.914)
**Number of Sons**	0.967	0.916
	(0.926 - 1.011)	(0.834 - 1.007)
**Number of Daughters**	0.963*	0.931
	(0.927 - 1.000)	(0.860 - 1.008)
**Care structure (ref: CaredbyOther)**		
**CaredbyDaughter**	0.772***	
	(0.665 - 0.898)	
**CaredbySon**	1.043	
	(0.942 - 1.156)	
**CaredbyBoth**	0.931	
	(0.805 - 1.077)	
**Care structure (ref: OnlyDaughterCare)**		
**OnlySonCare**		1.466**
		(1.144 - 1.879)
**Urban**	1.137**	1.229
	(1.036 - 1.249)	(0.991 - 1.525)
**Observations**	9,142	1,687
**LR chi2**	225.49	104.06
***Prob* > chi2**	0.0000	0.0000
**AIC**	18459.22	3598.712
***F*-test (*p*-value)**	0.0003	

Note: We use an ordinal logistic model, and the odds ratios (0Rs) are reported. The 95% confidence intervals are reported in the parentheses. Year and province dummies are included in all models, but their coefficients are not reported for brevity. The *P*-value of *F*-test for the equality of coefficients on the 'CarebyDaughter' and 'CarebySon' is reported at the end of the table.

*p < .05, **p < .01, ***p < .001.

Table A3: Ordinal logistic regression models for self-reported health and care provided by son and daughter: differentiating by region

	Model 1		Model 2	
	(1)	(2)	(3)	(4)
	Urban	Rural	Urban	Rural
**Male**	1.084	0.990	0.939	1.158
	(0.924 - 1.272)	(0.885 - 1.107)	(0.617 - 1.431)	(0.876 - 1.530)
**Age**	1.009*	1.001	1.012	1.017*
	(1.001 - 1.018)	(0.995 - 1.007)	(0.991 - 1.032)	(1.003 - 1.031)
**Marital status (ref. single or divorced)**				
**Married**	1.048	0.870	1.133	0.866
	(0.703 - 1.562)	(0.680 - 1.112)	(0.552 - 2.325)	(0.535 - 1.400)
**Widowed**	1.312	1.033	1.190	0.806
	(0.846 - 2.035)	(0.784 - 1.360)	(0.561 - 2.523)	(0.488 - 1.333)
**Primary Education**	0.768**	0.774***	1.050	0.858
	(0.653 - 0.904)	(0.686 - 0.874)	(0.708 - 1.555)	(0.623 - 1.183)
**Household Income**	1.012	1.024*	1.026	1.036
	(0.986 - 1.039)	(1.003 - 1.046)	(0.971 - 1.085)	(0.990 - 1.085)
**Working**	1.894***	1.617***	1.175	1.113
	(1.462 - 2.454)	(1.315 - 1.988)	(0.526 - 2.626)	(0.559 - 2.217)
**Number of Sons**	1.002	0.952	1.129	0.857**
	(0.927 - 1.083)	(0.902 - 1.005)	(0.943 - 1.351)	(0.764 - 0.962)
**Number of Daughters**	0.969	0.956	1.028	0.883*
	(0.906 - 1.037)	(0.913 - 1.002)	(0.891 - 1.185)	(0.800 - 0.974)
**Care structure (ref: CaredbyOther)**				
**CaredbyDaughter**	0.914	0.696***		
	(0.716 - 1.166)	(0.574 - 0.845)		
**CaredbySon**	1.149	1.004		
	(0.951 - 1.389)	(0.887 - 1.135)		
**CaredbyBoth**	0.801	1.006		
	(0.625 - 1.025)	(0.838 - 1.207)		
**Care structure (ref: OnlyDaughterCare)**				
**OnlySonCare**			1.085	1.594**
			(0.717 - 1.644)	(1.155 - 2.201)
**Observations**	2,893	6,249	559	1,128
**LR chi2**	116.66	158.18	54.13	83.54
***Prob* > chi2**	0.0000	0.0000	0.0433	0.0000
**AIC**	5900.187	12576.43	1263.322	2371.701
***F*-test (*p*-value)**	0.0937	0.0005		

Note: We use an ordinal logistic model, and the odds ratios (0Rs) are reported. The 95% confidence intervals are reported in the parentheses. Year and province dummies are included in all models, but their coefficients are not reported for brevity. The *P*-values of *F*-tests for the equality of coefficients on the 'CarebyDaughter' and 'CarebySon' are reported at the end of the table.

**p* < .05, ***p* < .01, ****p* < .001.

Table A4: Ordinal logistic regression models for self-reported health and care provided by son and daughter: differentiating by gender

	Model 1		Model 2	
	(1)	(2)	(3)	(4)
	Male	Female	Male	Female
**Age**	1.003	1.004	1.025	1.014*
	(0.995 - 1.012)	(0.997 - 1.010)	(1.000 - 1.051)	(1.001 - 1.027)
**Marital status (ref. single or divorced)**				
**Married**	1.109	0.842	1.655	0.723
	(0.761 - 1.615)	(0.654 - 1.084)	(0.748 - 3.660)	(0.453 - 1.153)
**Widowed**	1.369	1.000	1.569	0.726
	(0.885 - 2.117)	(0.757 - 1.321)	(0.679 - 3.627)	(0.445 - 1.183)
**Primary Education**	0.732***	0.791***	0.813	0.993
	(0.634 - 0.845)	(0.694 - 0.902)	(0.523 - 1.263)	(0.734 - 1.342)
**Household Income**	1.040**	1.008	1.047	1.024
	(1.012 - 1.070)	(0.988 - 1.029)	(0.971 - 1.129)	(0.984 - 1.066)
**Working**	2.018***	1.499***	2.089	0.927
	(1.594 - 2.553)	(1.203 - 1.869)	(0.838 - 5.208)	(0.491 - 1.751)
**Number of Sons**	1.014	0.940*	0.969	0.888*
	(0.942 - 1.091)	(0.890 - 0.993)	(0.800 - 1.174)	(0.794 - 0.992)
**Number of Daughters**	0.963	0.963	0.800**	0.984
	(0.904 - 1.025)	(0.918 - 1.010)	(0.682 - 0.939)	(0.896 - 1.081)
**Care structure (ref: CaredbyOther)**				
**CaredbyDaughter**	0.801	0.753**		
	(0.607 - 1.056)	(0.629 - 0.903)		
**CaredbySon**	1.018	1.066		
	(0.851 - 1.218)	(0.939 - 1.210)		
**CaredbyBoth**	0.835	0.976		
	(0.640 - 1.089)	(0.818 - 1.165)		
**Care structure (ref: OnlyDaughterCare)**				
**OnlySonCare**			2.046*	1.394*
			(1.173 - 3.567)	(1.050 - 1.851)
**Urban**	1.127	1.137*	0.995	1.302*
	(0.966 - 1.315)	(1.009 - 1.281)	(0.632 - 1.567)	(1.014 - 1.673)
**Observations**	3,335	5,807	419	1,268
**LR chi2**	138.20	138.26	65.86	81.44
***Prob* > chi2**	0.0000	0.0000	0.0017	0.0001
**AIC**	6709.542	11780.810	940.3634	2687.311
***F*-test (*p*-value)**	0.1206	0.0004		

Note: We use an ordinal logistic model, and the odds ratios (0Rs) are reported. The 95% confidence intervals are reported in the parentheses. Year and province dummies are included in all models, but their coefficients are not reported for brevity. The *P*-values of *F*-tests for the equality of coefficients on the 'CarebyDaughter' and 'CarebySon' are reported at the end of the table.

**p* < .05, ***p* < .01, ****p* < .001.

Table A5: Ordinal logistic regression models for self-reported health and care provided by son and daughter: differentiating by wealth

	Model 1		Model 2	
	(1)	(2)	(3)	(4)
	Wealthy	Less wealthy	Wealthy	Less wealthy
**Male**	1.086	1.016	0.927	1.179
	(0.887 - 1.329)	(0.916 - 1.126)	(0.586 - 1.466)	(0.898 - 1.549)
**Age**	0.993	1.006*	1.004	1.021**
	(0.983 - 1.004)	(1.001 - 1.012)	(0.981 - 1.027)	(1.007 - 1.035)
**Marital status (ref. single or divorced)**				
**Married**	1.291	0.834	1.101	0.961
	(0.752 - 2.216)	(0.665 - 1.046)	(0.407 - 2.975)	(0.618 - 1.495)
**Widowed**	1.542	0.989	1.292	0.848
	(0.855 - 2.781)	(0.767 - 1.275)	(0.450 - 3.707)	(0.535 - 1.344)
**Primary Education**	0.765*	0.740***	1.280	0.778
	(0.618 - 0.946)	(0.664 - 0.826)	(0.761 - 2.152)	(0.585 - 1.033)
**Household Income**	1.034*	1.011	1.068	1.013
	(1.001 - 1.069)	(0.992 - 1.030)	(0.999 - 1.142)	(0.971 - 1.057)
**Working**	1.962***	1.592***	2.974*	0.844
	(1.424 - 2.702)	(1.322 - 1.918)	(1.017 - 8.697)	(0.454 - 1.568)
**Number of Sons**	1.068	0.947*	1.046	0.858**
	(0.969 - 1.178)	(0.902 - 0.995)	(0.865 - 1.265)	(0.766 - 0.962)
**Number of Daughters**	1.051	0.947*	1.022	0.898*
	(0.967 - 1.143)	(0.908 - 0.989)	(0.870 - 1.200)	(0.817 - 0.986)
**Care structure (ref: CaredbyOther)**				
**CaredbyDaughter**	0.824	0.761**		
	(0.594 - 1.144)	(0.642 - 0.903)		
**CaredbySon**	1.061	1.025		
	(0.841 - 1.338)	(0.913 - 1.151)		
**CaredbyBoth**	0.970	0.903		
	(0.709 - 1.329)	(0.764 - 1.068)		
**Care structure (ref: OnlyDaughterCare)**				
**OnlySonCare**			1.538	1.445*
			(0.894 - 2.646)	(1.083 - 1.929)
**Urban**	1.053	1.127*	1.325	1.159
	(0.861 - 1.289)	(1.012 - 1.255)	(0.855 - 2.052)	(0.898 - 1.495)
**Observations**	1,812	7,330	414	1,273
**LR chi2**	83.76	171.13	88.02	85.10
***Prob* > chi2**	0.0001	0.0000	0.0000	0.0000
**AIC**	3909.53	14547.66	933.8019	2673.755
***F*-test (*p*-value)**	0.1478	0.0016		

Note: We use an ordinal logistic model, and the odds ratios (0Rs) are reported. The 95% confidence intervals are reported in the parentheses. Year and province dummies are included in all models, but their coefficients are not reported for brevity. The *P*-values of *F*-tests for the equality of coefficients on the 'CarebyDaughter' and 'CarebySon' are reported at the end of the table.

**p* < .05, ***p* < .01, ****p* < .001.

Table A6: Multinominal logistic regression models for the incidence of chronic disease and care provided by daughters and sons

	All		Wealthy		Less wealthy	
	**(1)**	**(2)**	**(3)**	**(4)**	**(5)**	**(6)**
	**Onset before**	**New onset**	**Onset before**	**New onset**	**Onset before**	**New onset**
**Male**	0.867*	0.919	0.849	0.808	0.870	0.948
	(0.760 - 0.989)	(0.818 - 1.032)	(0.636 - 1.133)	(0.620 - 1.054)	(0.749 - 1.010)	(0.833 - 1.080)
**Age**	1.011**	1.006	1.013	1.007	1.012**	1.007
	(1.004 - 1.019)	(1.000 - 1.013)	(0.999 - 1.028)	(0.994 - 1.020)	(1.003 - 1.021)	(0.999 - 1.014)
**Marital status (ref. single or divorced)**						
**Married**	0.978	1.307*	0.857	2.875**	1.003	1.139
	(0.728 - 1.312)	(1.005 - 1.700)	(0.433 - 1.696)	(1.402 - 5.896)	(0.721 - 1.396)	(0.855 - 1.518)
**Widowed**	0.899	1.081	0.814	2.348*	0.947	0.952
	(0.649 - 1.245)	(0.808 - 1.446)	(0.392 - 1.691)	(1.092 - 5.050)	(0.655 - 1.369)	(0.691 - 1.312)
**Primary Education**	1.384***	1.371***	1.275	1.384*	1.438***	1.378***
	(1.201 - 1.595)	(1.211 - 1.551)	(0.933 - 1.742)	(1.045 - 1.834)	(1.224 - 1.688)	(1.199 - 1.582)
**Household Income**	1.007	1.012	1.011	1.029	1.002	1.002
	(0.985 - 1.030)	(0.991 - 1.033)	(0.968 - 1.056)	(0.986 - 1.075)	(0.976 - 1.029)	(0.978 - 1.027)
**Working**	0.684**	0.677***	0.680	0.564**	0.679*	0.720**
	(0.528 - 0.886)	(0.556 - 0.824)	(0.407 - 1.136)	(0.374 - 0.850)	(0.502 - 0.920)	(0.574 - 0.903)
**Number of Sons**	1.013	0.995	0.931	0.989	1.028	0.988
	(0.954 - 1.076)	(0.942 - 1.050)	(0.818 - 1.059)	(0.876 - 1.115)	(0.959 - 1.102)	(0.930 - 1.051)
**Number of Daughters**	1.010	1.000	0.936	1.004	1.032	0.998
	(0.958 - 1.066)	(0.953 - 1.048)	(0.835 - 1.050)	(0.902 - 1.118)	(0.971 - 1.096)	(0.946 - 1.053)
**Care structure (ref: CaredbyOther)**						
**CaredbyDaughter**	1.132	1.315**	0.800	1.062	1.250	1.390**
	(0.911 - 1.406)	(1.093 - 1.583)	(0.505 - 1.267)	(0.706 - 1.596)	(0.975 - 1.604)	(1.127 - 1.714)
**CaredbySon**	1.070	1.133	0.943	1.057	1.106	1.172*
	(0.922 - 1.241)	(0.992 - 1.294)	(0.674 - 1.317)	(0.774 - 1.443)	(0.935 - 1.310)	(1.010 - 1.360)
**CaredbyBoth**	1.120	1.303**	0.863	1.285	1.245	1.299*
	(0.904 - 1.388)	(1.086 - 1.563)	(0.546 - 1.364)	(0.857 - 1.926)	(0.974 - 1.592)	(1.057 - 1.597)
**Urban**	1.025	1.053	0.871	1.217	1.065	1.002
	(0.894 - 1.175)	(0.934 - 1.187)	(0.652 - 1.166)	(0.932 - 1.590)	(0.910 - 1.245)	(0.875 - 1.148)
**Constant**	0.952	0.431**	1.489	0.170**	0.824	0.516*
	(0.502 - 1.807)	(0.242 - 0.768)	(0.379 - 5.852)	(0.0453 - 0.639)	(0.395 - 1.718)	(0.268 - 0.991)
**Observations**	9,733	9,733	2,024	2,024	7,709	7,709
**LR chi2**	2829.36		593.42		2319.77	
***Prob* > chi2**	0.0000		0.0000		0.0000	
**AIC**	18496.7		3950.094		14624.91	

Note: We use a multinominal logistic model, and the relative risk ratios (RRR) are reported. The 95% confidence intervals are reported in the parentheses. Year and province dummies are included in all models, but their coefficients are not reported for brevity.

*p < .05, **p < .01, ***p < .001.
